# The Time Course of Dynamic Computed Tomographic Appearance of Radiation Injury to the Cirrhotic Liver Following Stereotactic Body Radiation Therapy for Hepatocellular Carcinoma

**DOI:** 10.1371/journal.pone.0125231

**Published:** 2015-06-11

**Authors:** Tomoki Kimura, Shigeo Takahashi, Ippei Takahashi, Ikuno Nishibuchi, Yoshiko Doi, Masahiro Kenjo, Yuji Murakami, Yohji Honda, Hiroshi Aikata, Kazuaki Chayama, Yasushi Nagata

**Affiliations:** 1 Department of Radiation Oncology, Graduate School of Biomedical Sciences, Hiroshima University, Hiroshima, Japan; 2 Department of Radiation Oncology, Kagawa University Hospital, Takamatsu, Japan; 3 Department of Medicine and Molecular Science, Division of Frontier Medical Science, Programs for Biomedical Research, Graduate School of Biomedical Sciences, Hiroshima University, Hiroshima, Japan; Kanazawa University, JAPAN

## Abstract

This study aimed to evaluate the dynamic computed tomographic (CT) appearance of focal radiation injury to cirrhotic liver tissue around the tumor following stereotactic body radiation therapy (SBRT) for hepatocellular carcinoma (HCC). Seventy-seven patients with 92 HCCs were observed for >6 months. Sixty-four and 13 patients belonged to Child–Pugh class A and B, respectively. The median SBRT dose was 48 Gy/4fr. Dynamic CT scans were performed in non–enhanced, arterial, portal, and venous phases. The median follow-up period was 18 months. Dynamic CT appearances were classified into 3 types: type 1, hyperdensity in all enhanced phases; type 2, hypodensity in arterial and portal phases; type 3, isodensity in all enhanced phases. Half of the type 2 or 3 appearances significantly changed to type 1, particularly in patients belonging to Child–Pugh class A. After 3–6 months, Child–Pugh class B was a significant factor in type 3 patients. Thus, dynamic CT appearances were classified into 3 patterns and significantly changed over time into the enhancement group (type 1) in most patients belonging to Child–Pugh class A. Child–Pugh class B was a significant factor in the non–enhancement group (type 3).

## Introduction

Radiation-induced liver disease (RILD) is an adverse effect pathologically characterized as a veno-occlusive disease (VOD) [1] that often exhibits severe or fatal complications following conventional radiation therapy for large hepatic volumes. However, recent advances in imaging and radiation techniques provide high radiation doses to conform to focal hepatocellular carcinoma (HCC), and several studies have described good treatment results for stereotactic body radiation therapy (SBRT) or particle therapy without severe clinical signs of RILD [2–5]. Despite the decreasing incidence of RILD, focal radiation injury to normal or cirrhotic liver tissue around the tumor has been observed on follow-up dynamic computed tomography (CT). The CT appearance of focal radiation injury reflects the irradiated area of the normal liver, therefore it is important to correctly investigate the imaging finding following SBRT because HCC patients may require additional multiple therapies in future. The CT appearance of radiation injury has also been reported, and we have often observed that injury appearance following SBRT changed with time and differs from that following conventional radiotherapy [6–8]. Few studies have investigated the relationship between the change in CT appearance over time following SBRT and related factors or adverse effects. We have also observed that the CT appearance of tumor response following SBRT differs from that following conventional radiotherapy [9]; however, we focused on normal or cirrhotic liver injury in the present study.

The present study aimed to evaluate the dynamic CT appearance of focal radiation injury to cirrhotic liver tissue around the tumor following SBRT for HCC and to investigate the relationship between CT appearance and clinical features.

## Materials and Methods

### Patient background

Seventy-seven patients with 92 HCCs observed by dynamic CT for more than 6 months were included in this study. Eight patients underwent simultaneous SBRT for 2 HCC lesions each, whereas 7 patients with solitary HCC lesions were treated at different times. Most patients had previously undergone surgery or ablation therapies, and SBRT was recommended if these options were limited by technical difficulties or if the patient was inoperable or refused surgery. The study protocol was approved by the Human Ethics Review Committee of Hiroshima University and a signed consent form was obtained from each subject. The median time interval between the initial treatment and SBRT was 25 months (range, 0–144 months). Table 1 summarizes the patient characteristics, including age, gender, Eastern Cooperative Oncology Group, performance status, type of viral infection, Child–Pugh class and scoring, primary tumor location and previous treatments. Most patients were carriers of hepatitis B virus (HBV) or hepatitis C virus (HCV) (80.5%), and 13 patients were classified into Child–Pugh class B (16.7%).

**Table 1:** Patients Background (77 patients with 92 HCCs) .

**Age**		49–90 (median:71)
**Tumor size**		3–54 mm (median:19 mm)
**Gender**	male	49 patients
	female	28 patients
**Performance status (PS)**	0	74 patients
	1	3 patients
**Type of viral infection** *	HBV	6 patients
	HCV	62 patients
	NBNC	9 patients
**Child-Pugh class**	A	64 patients
	B	13 patients
**Child-Pugh score**	5	46 patients
	6	16 patients
	7	10 patients
	8≧	5 patients
**Tumor location**	S1	2 lesion
	S2	1 lesion
	S3	8 lesions
	S4	16 lesions
	S5	11 lesions
	S6	9 lesions
	S7	19 lesions
	S8	26 lesions
**Previous treatment** surgery		29 patients
	RFA**	25 patients
	PEI^#^	12 patients
	TACE^$^	71 patients

Abbreviation:

* HBV; hepatitis B virus,HCV; hepatitis C virus, NBNC; non-hepatitis B non-hepatitis C

** RFA; radiofrequency ablation,

^#^ PEI; percutaneous ethanol injection

^$^ TACE; transcatheter arterial chemoembolization

HCC was diagnosed by its characteristic findings on dynamic CT or angiography combined with CT, which showed early enhancement at the arterial phase and hypodensity at the portal–venous phase in most patients. These CT findings were not observed in 6 lesions, and HCC was diagnosed histologically.

### Treatment procedure

Prior to SBRT, 71 patients with 85 HCCs underwent transcatheter arterial chemoembolization (TACE) with iodized lipiodol. An anticancer drug, such as epirubicin, cisplatin mixed with lipiodol (7–70 mg/body at 10 mg/mL lipiodol) or miriplatin mixed with lipiodol (20–80 mg/body at 20 mg/mL lipiodol) was injected into the hepatic artery that fed a segment or subsegments containing the target tumor. The selected dose was based on the tumor size and liver function. Embolization was subsequently induced with a small amount of gelatin sponge particles until the flow into the feeding artery decreased markedly. The median time interval between TACE and SBRT was 2 months (range, 1–7 months).

SBRT was performed using a 3-dimensional (3D) conformal method that delivered a single high dose of radiation to the tumor. A vacuum cushion (Vac-Lok; CIVCO, Kalona, IA, USA) was used to fix the patient’s body. Respiratory motion was evaluated by X-ray simulation, and if it was more than 5 mm, the voluntary breath-hold method was used with a spirometer to coordinate respiratory motion or an Abches (APEX Medical, Tokyo, Japan), which is a device that allows the patient to self-control the respiratory motion of the chest and abdomen. Patients held their breath during the end-expiration phase because the interbreath-hold reproducibility of the organ position was superior to that of the end-inspiration phase [10]. This method was used in 73 patients with 87 lesions. The free-breathing method was used in 2 patients with 3 lesions, and respiratory–gating with the Real-time Position Management system (Varian Medical Systems, Palo Alto, CA, USA) was used in 2 patients with 2 lesions. For simulation, dynamic CT scans (Lightspeed QX/I; GE Medical Systems, Waukesha, WI, USA) using bolus injection of non-ionic iodinated contrast material (100 ml at a flow of 3 mL/s) were obtained in 4 phases, which included non-enhanced and contrast-enhanced scans in the arterial, portal, and venous phases. CT volume data in the arterial phase were transferred to a 3D treatment planning system (Pinnacle^3^ ver. 9.0, Phillips Medical Systems, Fitchburg, WI, USA). The gross tumor volume (GTV) was determined as the tumor volume that contained remaining lipiodol from TACE and early enhancement in the arterial phase of dynamic CT. A clinical target volume (CTV) margin of 3 mm was usually added to GTV for subclinical invasion. A planning target volume (PTV) margin of 5–8 mm was usually added to allow reproducibility of respiratory motion and CTV setup error. Eight noncoplanar ports were selected in all patients, including 4 or 5 coplanar beams and 3 or 4 noncoplanar beams in a direction that avoided the stomach, intestine, gallbladder and spine, if possible. The prescribed doses and fractionations were 60 Gy/8 fractions in 17 lesions, 50 Gy/5 fractions in 4 lesions, 40 Gy/4 fractions in 3 lesions, and 48 Gy/4 fractions in 68 lesions. The treatment involved 6–10 MV photons from a linear accelerator (CLINAC 2300 C/D or iX, Varian Medical Systems, Palo Alto, CA, USA) that delivered 600 monitor units/ min to reduce the breath-holding duration to 15 s or less for each treatment field.

### Evaluation

Follow-up dynamic CT scans were obtained at 3-month intervals following SBRT. A multi-detector row helical CT scanner (Light Speed Ultra 16 or Light Speed VCT, GE, Milwaukee, WI, USA) with a reconstructed slice width of 5 mm and a slice interval of 5 mm was used to perform CT examination. The scanning parameters were as follows: 120 kV, Auto mA (noise index 10), 5-mm section thickness, 1.375-beam pitch and 0.7 (Light Speed Ultra 16) and 0.4 (Light Speed VCT) rotation speed. Images were obtained in 4 phases, namely before contrast enhancement, arterial, portal and venous phase after injection using an automatic injector of 100–150 mL (600mgI/kg) of nonionic iodinated contrast agent (the iodine concentration; 350mgI/ml) at a rate of 4 mL/s. An automatic bolus-tracking program was used to time the start of scanning for each phase after contrast injection. Monitoring was performed at the L1 vertebral body level. The region-of-interest cursor (0.8–2.0 cm^2^) was placed in the abdominal aorta. The trigger threshold level was set at 200 HU. The arterial, portal and venous phase scanning was initiated at 15–17, 45–47, and 145–147 s after triggering with the multi-detector row helical CT scanner. A soft-tissue window (level, 40 HU; width, 200 HU) was used to evaluate the dynamic CT appearance, and the appearance of each of the 92 lesions was confirmed by consensus between 1 of the authors (T.K.) and 2 radiologists. For determination of irradiated area, we compared with dose distribution of planning dynamic CT and follow-up dynamic CT.

We evaluated following two issues.

1. Dynamic CT appearance patterns of hepatic radiation injuries and time course
2. We evaluated the dynamic CT appearance of hepatic radiation injury over various time courses, such as within 3 months, 3–6 months and 6–12 months. All the lesions were followed up for at least 6 months; however 14 lesions could not be followed–up for 12 months because of death or the need for follow-up using magnetic resonance imaging for 6–12-months.
3. Relationship between the dynamic CT appearance of hepatic radiation injury and clinical features for specific time periods
4. We also evaluated the relationship between injury appearance and clinical features or clinical results such as adverse effects. Treatment-related toxicities were evaluated by the Common Terminology Criteria for Adverse Events (CTCAE) ver.4. 0. The median follow–up period at the time of evaluation was 18 months (range, 6–49 months).

### Statistical methods

Univariate and multivariable analysis were performed using the Mantel–Haenzel χ^2^ or t–tests and logistic regression for comparison of statistical significance. StatMate for Windows (StatMate ver 4.01; ATMS, Tokyo, Japan) was used to perform all statistical analyses. Statistical significance was defined as p < 0.05.

## Results and Discussion

### Dynamic CT appearance patterns of hepatic radiation injuries and time course

**Fig 1:**
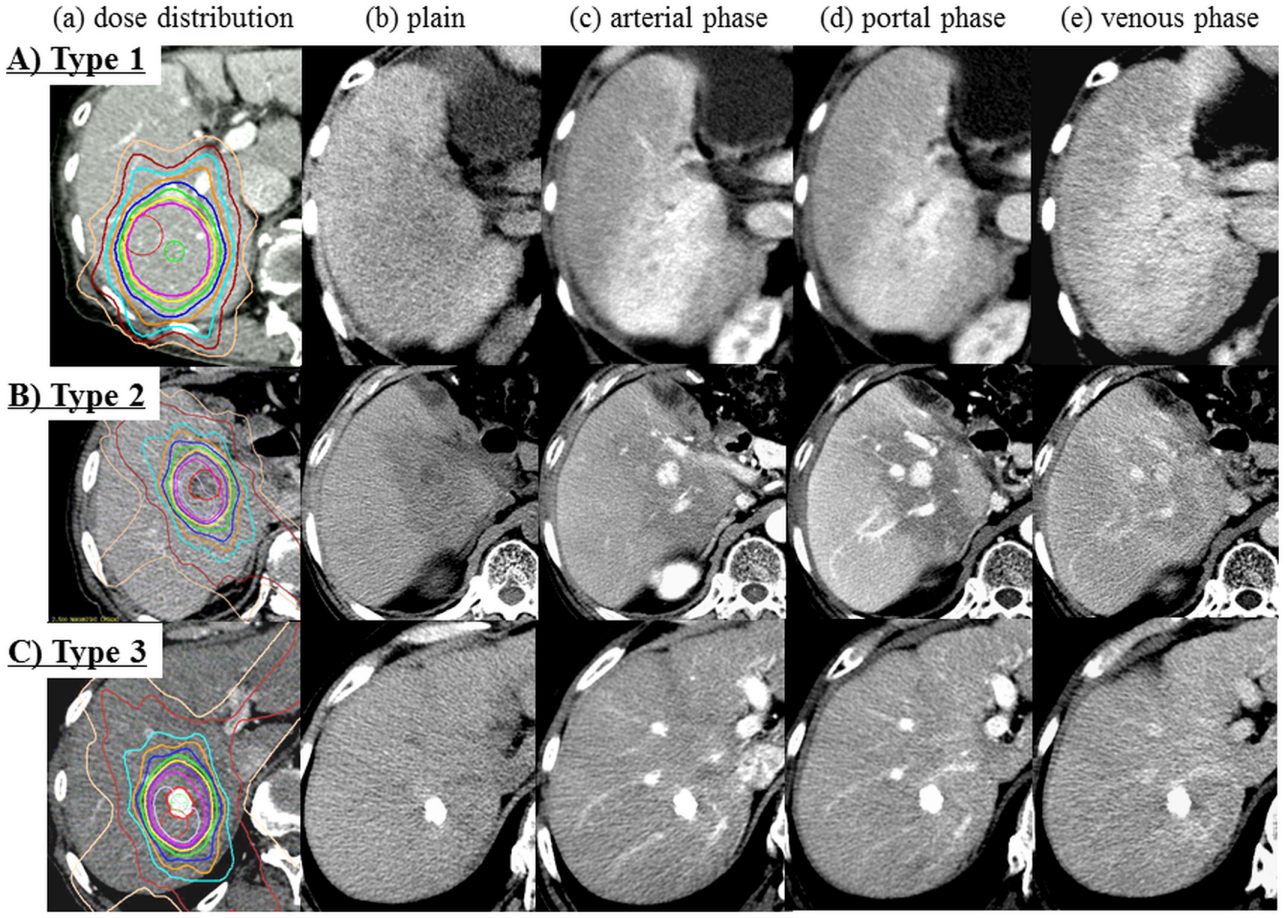
The dynamic CT appearance of focal liver injury following SBRT for HCC was classified into 3 types. **A**) Type 1 (case 12, 10 months following SBRT). a) Dose distribution b) Plain c) Arterial phase d) Portal phase e) Venous phase. Hypodensity in plain CT and hyperdensity in all enhanced phases. B) Type 2 (case 39, 2 months following SBRT). a) Dose distribution b) Plain c) Arterial phase d) Portal phase e) Venous phase. Hypodensity in the arterial and portal phases and isodensity in the venous phase. C) Type 3 (case 51, 2 months following SBRT). a) Dose distribution b) Plain c) Arterial phase d) Portal phase e) Venous phase. Isodensity in the enhanced phases.

The dynamic CT appearance of hepatic radiation injury was classified into the following 3 types: type 1, hyperdensity in all enhanced phases (Fig 1A); type 2, hypodensity arterial, and portal phases, isodensity in venous phase and isodensity in the venous phase (Fig 1B); and type 3, isodensity in all enhanced phases (Fig 1C). These types may be divided into 2 main subtypes: type 1, with enhancement and type 2 or 3, with nonenhancement.

**Fig 2:**
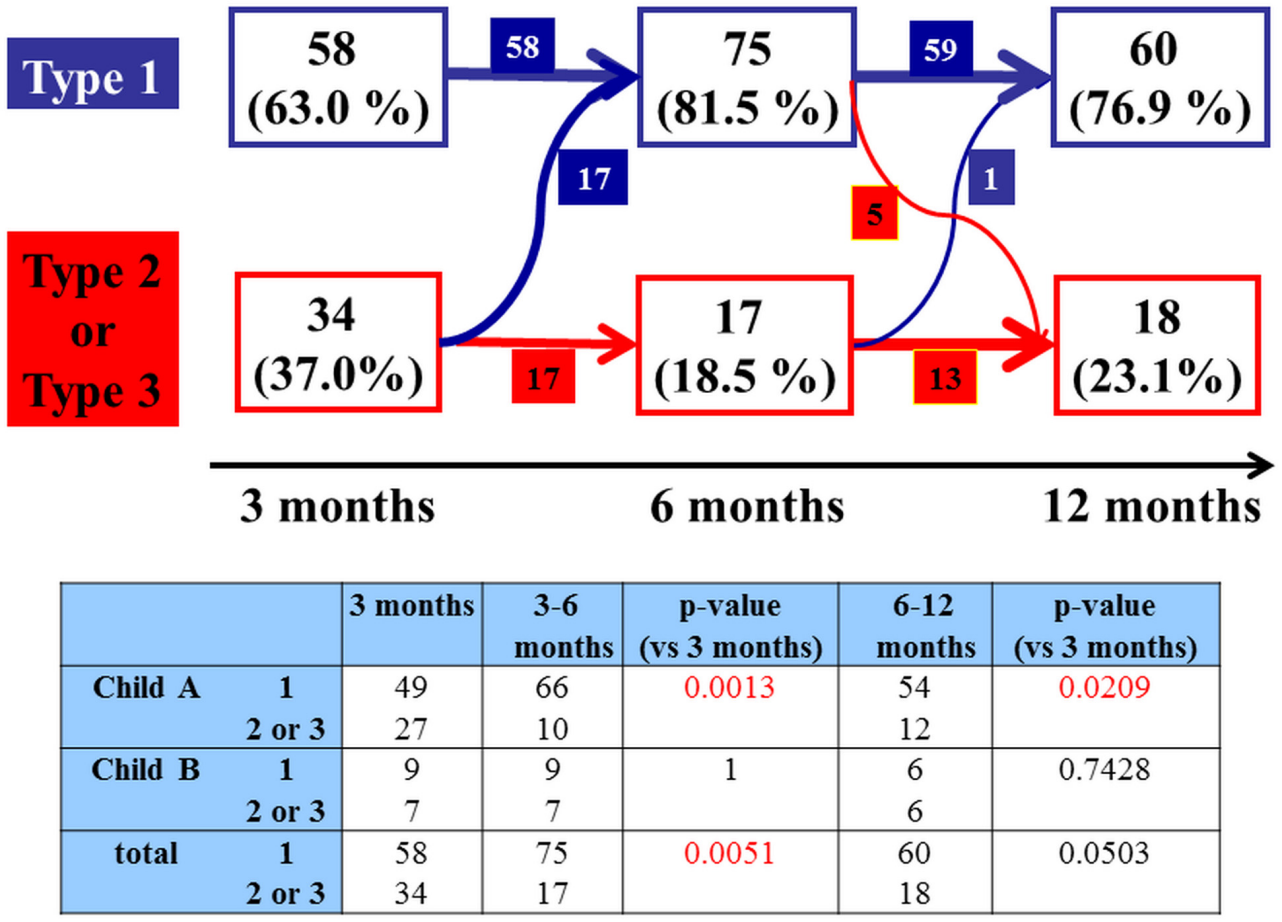
The time course of dynamic CT appearance of radiation injury to liver according to Child-Pugh class. Half of type 2 or 3 lesions changed to type 1 at 3–6 months following SBRT, and the change was significant (p = 0.0051). According to the Child–Pugh class, more than half of the type 2 or 3 lesions of Child–Pugh class A changed into type 1 throughout the time course, and this was a significant change (p = 0.0013 at 3–6 months, p = 0.0209 at 6–12 months). On the other hand, the type 2 or 3 lesions of Child–Pugh class B tended to remain unchanged.

**Fig 3:**
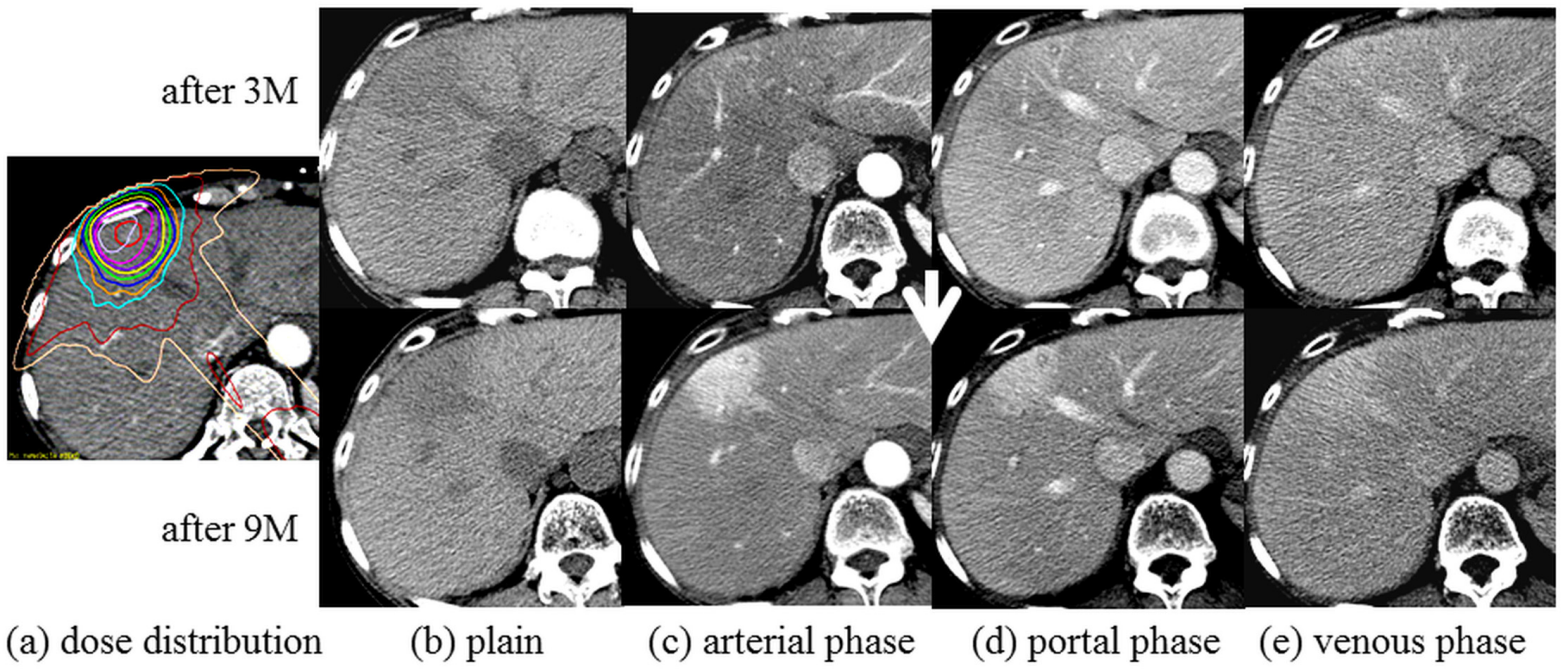
A typical case belonging to Child–Pugh class A that changed from type 3 after 3 months to type 1 after 9 months (case 36). Dose distribution b) Plain c) Arterial phase d) Portal phase e) Venous phase.

The type of CT appearance changed during the follow-up period. Within 3 months, type 1 was observed in 58 lesions (63.0%), type 2 in 17 lesions (18.5%) and type 3 in 17 lesions (18.5%). Within 3–6 months, type 1 was observed in 75 lesions (81.5%), type 2 in 11 lesions (12.0%) and type 3 in 6 lesions (6.5%). Within 6–12 months, during which only 78 lesions were followed up to 12 months following SBRT, type 1 was observed in 60 lesions (76.9%), type 2 in 8 lesions (10.3%) and type 3 in 10 lesions (12.8%). The previous therapy may have effects on the dynamic CT appearance, however there were no significant difference in each enhancement pattern, such as type 1 to 3 in this study (Table 2). Fig 2 shows the changes in types of CT appearance during the follow–up period. Half of type 2 or 3 lesions changed to type 1 at 3–6 months following SBRT, and the change was significant (p = 0.0051). According to the Child–Pugh class, more than half of the type 2 or 3 lesions of Child–Pugh class A changed into type 1 throughout the time course, and this was a significant change (p = 0.0013 at 3–6 months, p = 0.0209 at 6–12 months). On the other hand, the type 2 or 3 lesions of Child–Pugh class B tended to remain unchanged. Fig 3 shows a typical case belonging to Child–Pugh class A that changed from type 3 to type 1.

**Table 2:** Background of dynamic CT appearance of radiation injury according to previous therapy.

Previous therapies		3 months				3–6 months				6–12 months			
		Type 1	Type 2	Type 3	p-value	Type 1	Type 2	Type 3	p-value	Type 1	Type 2	Type 3	p-value
**TACE** *	**+**	54	16	15	0.3176	68	11	6	0.0961	53	8	10	0.066
	**-**	4	1	2		7	0	0		7	0	0	
**Surgery**	**+**	24	6	8	0.4446	31	5	2	0.4765	25	3	6	0.2284
	**-**	34	11	9		44	6	4		35	5	4	
**Ablative therapies**	**+**	23	9	3	0.188	29	4	2	0.3938	25	4	2	0.2131
	**-**	35	8	14		46	7	4		35	4	8	

* TACE; transcatheter arterial chemoembolization

### Relationship between the dynamic CT appearance of hepatic radiation injury and clinical features for specific time periods

Table 3 shows the results of univariate and multivariable analysis of the dynamic CT appearance of hepatic radiation injury for various time periods and the clinical features between type 3 or nontype 3 at 3–6 months, including the Child–Pugh class, gender, age, total dose, PTV, tumor location, history of resection, duration of initial treatment and adverse effects. The Child–Pugh class was a significant factor in type 3 or nontype 3 (p < 0.0001). Ten patients with 11 lesions (13.0%) developed grade 3 toxicities such as elevated bilirubin and aspartate aminotransferase/alanine aminotransferase (AST/ALT) levels and portal thrombosis, and these toxicities at 3–6 months were also significant factors in type 3 appearances (p = 0.003). In multivariable analysis, the Child–Pugh class was a only significant factor in type 3 or nontype 3 (p = 0.0005).

**Table 3:** Univariate and Multivariate Analysis between the dynamic CT appearance of radiation injury to the liver and clinical features on Type 3 or Non-Type 3.

		3–6 months		P-value	P-value
		Type 3	Non-Type 3	Uni^#^	Multi^##^
**Child-Pugh class**	A	0	76	<0.0001	0.0005
	B	6	10		
**Gender**	male	5	53	0.2869	-
	female	1	33		
**Age**	>75	1	33	0.2869	-
	≦75	5	53		
**Total dose**	>48Gy	2	19	0.5259	-
	≦48Gy	4	67		
**PTV**	>25cc	1	35	0.2436	-
	≦25cc	5	51		
**Liver V20** * **(Liver-PTV)**	>10%	3	42	0.9561	-
	≦10%	3	44		
**Tumor location**	periferal	4	70	0.3792	-
	central	2	16		
**History of resection**	+	2	36	0.6817	-
	-	4	50		
**Duration from first treatment**	>12 months	3	57	0.4182	-
	≦12 months	3	29		
**Adverse effects** **	Grade 1 or 2	3	78	0.003	0.5666
	Grade 3	3	8		

Abbreviation:

*V20: the percentage of the liver excluding PTV volume exceeding 20 Gy

** Adeverse effects were evaluated CTCAE ver.4.0.

^#^ uni: univariate analysis by the Mantel-Haenzel χ2 or t tests

^##^ Multi: univariate analysis by the Mantel-Haenzel χ2 or t tests

## Discussion

Several authors have described dynamic CT appearances of focal liver reactions following radiation therapy including SBRT [6– 8]. Limited to SBRT, Herfarth et al. reported the CT appearance of liver malignancies, which were mostly metastatic tumors, following single–high–dose SBRT [6, 7]. Three different appearance types of the reaction could be defined on the basis of the liver density in the portal–venous and late phases after contrast agent administration. In other words, type 1 showed hypodensity in the portal–venous phase and isodensity in the late contrast phase, type 2 showed hypodensity in the portal–venous phase and hyperdensity in the late contrast phase, and type 3 showed isodensity/ hyperdensity in the portal–venous phase and hyperdensity in the late contrast phase. In addition, Sanuki-Fujimoto described the CT appearance of focal liver reactions following SBRT for HCC with cirrhosis and classified 3 types, which were identified in the pre-contrast, arterial, and portal–venous phases as iso/iso/iso (type A), low/iso/iso (type B) and low/iso(or high)/high (type C) [8]; type C was the most common appearance in patients. Comparison of the results revealed that types 1 and 2 reported by Herfarth et al. and types A and B reported by Sanuki-Fujimoto correspond to types 2 and 3 reported in the present study, whereas type 3 reported by Herfarth et al. and Type C reported by Sanuki-Fujimoto appear to correspond to type 1 reported in the present study.

In the present study, although there were no pathological findings associated with these types of CT appearances, we considered the hemodynamic mechanism underlying findings of these 3 types. Our type 1, which included most patients belonging to Child–Pugh class A, could be based on the histopathological features of VOD, which has been recognized as hepatic radiation injury [11–14]. In other word, in the patients with preserved hepatic function, hepatic vein occlusion causes drainage from portal vein and compensatory increase of inflow from hepatic artery, resulting in showing hyperdensity in arterial phase. Olsen et al. described VOD with marked sinusoidal congestion and venous damage in 2 patients who underwent exploratory surgery following SBRT [15]. Willemart et al. reported that the appearance of hypodensity in the portal–venous phase that becomes hyperdense in the delayed phase can be explained by decreased vascular perfusion and reduced hepatic venous drainage with subsequent stasis of the contrast medium [11]. The hemodynamic mechanism of our type 3 could be basically similar to that of our type 1; however, our type 3 included most patients belonging to Child–Pugh class B, in whom decrease hepatic arterial and portal perfusion due to liver cirrhosis could not make it possible to enhance the irradiated area by a contrast agent. The portal perfusion is usually decreased as a result of the increase in intrahepatic vascular resistance and compensatory arterial perfusion fraction is increased in cirrhotic liver. However, in cirrhosis, the increase in arterial perfusion is often not sufficient to maintain total liver perfusion owing to high extrahepatic portosystemic shunting [16–18]. For patients with Child-Pugh class B, poor blood flow in both the portal vein and artery results in a decreased enhancement effect. Type 2 may be rather close to type 3, however the enhancement effect of background liver may be maintained in type2 compared to type 3. Therefore, the irradiated area is observed as hypodensity compared to the background liver in arterial and portal phase. In other words, because of congestion by hepatic vein occlusion, the inflow from portal vein and hepatic artery may be delayed, resulting in showing hypodensity in arterial and portal phase. Poor venous drainage with earlier pooling for Child–Pugh class A liver injury and poor blood flow in both the portal vein and artery in cirrhosis, similar to that observed in Child–Pugh class B, resulted in a decreased enhancement effect. Although most patients belonging to Child–Pugh class A should change to type 1 according to these theories, this was not necessarily the case. Most patients in the present study underwent TACE, and this fact may be explained by the effects of spontaneous arterial occlusion by TACE.

It is well known that the CT appearance following SBRT changes over time [6– 8]. Herfarth et al. reported that the median onset time for observable reactions was 1.8 months following single high–dose SBRT in patients with metastatic liver tumors. They also reported that while reactions showing hypodensity in the portal–venous phase usually appeared earlier, reactions showing hyperdensity in the portal–venous and late contrast phases appeared later during the follow–up [6, 7]. Sanuki-Fujimoto reported that no difference was observed in the timing of appearance between the enhancement and nonenhancement groups in HCC patients with liver cirrhosis. They suggested that the reason why the background liver functions differed from those in the study of Herfarth et al. was that most patients had metastatic liver tumors [8]. In the present study, most cases underwent a significant change to type 1, (enhancement group, p = 0.0013 at 3–6 months and 0.0209 at 6–12 months; Fig 2) and the time course for this change to the hyperdense type was similar to that in the study of Herfarth et al. in patients belonging to Child–Pugh class A. However, when considering only patients belonging to Child–Pugh class B, most type 2 or 3 (nonenhanced type) cases did not change over time, which differed from the result of the study of Herfarth et al. Assessment of the dynamic CT appearance of hepatic radiation injury and clinical features over time showed that type 1 significantly increased in patients belonging to Child–Pugh class A (Fig 2) after 3 months. In contrast, the percentage of nonenhanced types (types 2 and 3), particularly type 3, significantly increased in patients belonging to Child–Pugh class B over time (Fig 2, Table 3). These results were similar to those of the study of Sanuki-Fujimoto, which showed that most Child–Pugh class A lesions had hyperdense appearances, whereas most Child–Pugh class B lesions were nonenhanced; this difference was significant [8]. In addition, the present study showed that this trend was significant over time. Based on these results, we should consider that CT appearances of focal radiation injuries to cirrhotic liver tissues, particularly those belonging to Child–Pugh class B, differ from those to normal liver tissues. Sanuki-Fujimoto concluded that the CT appearance of the cirrhotic liver following SBRT for HCC was related to the background liver function and should not be misread as a recurrence of HCC [8]. Other imaging modalities such as gadoxetate disodium- enhanced hepatic MRI (EOB- MRI) must be required to correctly determine the irradiated area for avoiding severe toxicities, particularly in patients belonging to Child–Pugh class B whose CT appearance is classified into nonenhanced types. Motosugi et al suggested that it can be said that EOB MRI may be applied to evaluate hepatic function, especially for “partial hepatic function”. Moreover they demonstrated that whether liver enhancement is sufficient should be determined using a quantitative criterion—liver-spleen contrast of more than 1.5 [19].

In addition, we evaluated the relationship between the dynamic CT appearances of radiation injury to cirrhotic liver and adverse effects over time. In type 3, which did not show enhancement in all phases, grade 3 adverse effects were significant. These results can be explained by the high percentage of Child–Pugh class B patients in the nonenhanced group, particularly for type 3.

We are aware that this study, because of its retrospective nature, has certain limitations such as the low number of patients, particularly those belonging to Child–Pugh B, the absence of pathological findings for these types of CT appearances and the effects of previous treatment. SBRT can still be considered to be an alternative to surgery, ablation, and TACE when these therapies fail, and most of our patients had previously undergone these therapies, which may have influenced the CT appearance of hepatic radiation injury following SBRT. We are currently planning a prospective study to address the above mentioned points.

In conclusion, the dynamic CT appearance of focal liver injury following SBRT for HCC was classified into 3 types. Most cases showed significant changes over time to the enhancement group (type 1, appearance in the patients with Child–Pugh class A after 3 months). However, the CT appearance particularly did not change significantly over time in patients in the nonenhancement group (type 3) and Grade 3 adverse effects were significantly worse in this type. Sanuki et al suggested EOB-MRI was useful for detecting threshold dose after SBRT for HCC with chronic liver disease, and the use of the threshold dose will help to predict potential loss of liver tissue after SBRT [20]. Thus, it is important to investigate the correct irradiated area from EOB- MRI appearance in this type for additional therapies. The other imaging studies are required to correctly determine the irradiated areas in these patients for additional therapies in future.

The dynamic CT appearance of tumor response after SBRT is also various [9], and it is sometime difficult to distinguish from the CT appearance of normal liver injury. This study suggested that it will be useful to understand these various imaging patterns of normal liver injury for evaluation of tumor response after SBRT.

## Conclusions

Dynamic CT appearances were classified into 3 patterns and significantly changed over time into the enhancement group (type 1) in most patients belonging to Child–Pugh class A. Child–Pugh class B was a significant factor in the non–enhancement group (type 3).

## Supporting Information

S1 Table Supplementary Table of raw data in this study. This table shows the clinical features, such as the Child–Pugh class, gender, age, total dose, PTV, tumor location (centrally located), history of resection, duration of initial treatment and adverse effects (Grade 3), according to type 1–3.
Type 1, hyperdensity in all enhanced phasesType 2, hypodensity arterial, and portal phases, isodensity in venous phase and isodensity in the venous phaseType 3, isodensity in all enhanced phases.
(PDF)

S2 Table Supplementary Table of Tables 1 and 3 in this study. The title of Table 1 is “Patient Background (77 patients with 92 HCCs)”. This table shows the patients backgrounds, including age, gender, PS, type of viral infection, Child-Pugh class, Child-Pugh score, tumor size, tumor location and previous treatment. The title of Table 3 is “Univariate and Multivariate Analysis between the dynamic CT appearance of radiation injury to the liver and clinical features on Type 3 or Non-Type 3”. This table shows the results of univariate and multivariable analysis of the dynamic CT appearance of hepatic radiation injury for various time periods and the clinical features between type 3 or nontype 3 at 3–6 months, including the Child–Pugh class, gender, age, total dose, PTV, tumor location, history of resection, duration of initial treatment and adverse effects. The Child–Pugh class and adverse effects were significant factors in type 3 or nontype 3 (p < 0.0001, p = 0.003, respectively). In multivariable analysis, the Child–Pugh class was a only significant factor in type 3 or nontype 3 (p = 0.0005).
(PDF)

S3 Table Supplementary Table of Table 2 in this study. The title of Table 2 is “Background of dynamic CT appearance of radiation injury according to previous therapy”. This table shows there were no significant difference in each enhancement pattern, such as type 1 to 3 in this study.
(PDF)

S1 Fig Supplementary Figure of Fig 1A in this study. The title of Fig 1A is “The dynamic CT appearance of focal liver injury following SBRT for HCC was classified into 3 types. A) Type 1 (case 12, 10 months following SBRT)”. a) Dose distribution b) Plain c) Arterial phase d) Portal phase e) Venous phase.
(TIF)

S2 Fig Supplementary Figure of Fig 1B in this study. The title of Fig 1B is “The dynamic CT appearance of focal liver injury following SBRT for HCC was classified into 3 types. B) Type 2 (case 39, 2 months following SBRT)”. a) Dose distribution b) Plain c) Arterial phase d) Portal phase e) Venous phase.
(TIF)

S3 Fig Supplementary Figure of Fig 1C in this study. The title of Fig 1C is “The dynamic CT appearance of focal liver injury following SBRT for HCC was classified into 3 types. C) Type 3 (case 51, 2 months following SBRT)”. a) Dose distribution b) Plain c) Arterial phase d) Portal phase e) Venous phase.
(TIF)

S4 Fig Supplementary Figure of Fig 2 in this study. The title of Fig 2 is “The time course of dynamic CT appearance of radiation injury to liver according to Child-Pugh class”. The details of this figure was shown in Figure legends “Fig 2”.
(TIF)

S5 Fig Supplementary Figure of Fig 3 in this study. The title of Fig 3 is “A typical case belonging to Child–Pugh class A that changed from type 3 after 3 months to type 1 after 9 months (case 36).” Dose distribution b) Plain c) Arterial phase d) Portal phase e) Venous phase.
(TIF)

## References

[1] Sempoux C, Horsmans Y, Geubel A, Fraikin J, Van Beers BE, Gigot J, et al (1997) Severe radiation-induced liver disease following localized radiation therapy for biliopancreatic carcinoma: Activation of hepatic stellate cells as an early event. Hepatology 26: 128–34. 9214461 10.1002/hep.510260117

[2] Andolino DL, Johnson CS, Maluccio M, Kwo P, Tector AJ, Zook J, et al (2011) Stereotactic body radiotherapy for primary hepatocellular carcinoma. Int J Radiat Oncol Biol Phys 81: e447–53. 10.1016/j.ijrobp.2011.04.011 21645977

[3] Takeda A, Takahashi M, Kunieda E, Takeda T, Sanuki N, Koike Y, et al (2008) Hypofractionated stereotactic radiotherapy with and without transarterial chemoembolization for small hepatocellular carcinoma not eligible for other ablation therapies: Preliminary results for efficacy and toxicity. Hepatol Res 38: 60–9. 17506837 10.1111/j.1872-034X.2007.00084.x

[4] Fukumitsu N, Sugahara S, Nakayama H, Fukuda K, Mizumoto M, Abei M, et al (2009) A prospective study of hypofractionated proton beam therapy for patients with hepatocellular carcinoma. Int J Radiat Oncol Biol Phys 74: 831–6. 10.1016/j.ijrobp.2008.10.073 19304408

[5] Kato H, Tsujii H, Miyamoto T, Mizoe JE, Kamada T, Tsuji H, et al (2004) Results of the first prospective study of carbon ion radiotherapy for hepatocellular carcinoma with liver cirrhosis. Int J Radiat Oncol Biol Phys 59: 1468–76. 15275734 10.1016/j.ijrobp.2004.01.032

[6] Herfarth KK, Hof H, Bahner ML, Lohr F, Hoss A, Kaick G, et al (2003) Assessment of focal liver reaction by multiphasic CR after stereortactic single-dose radiotherapy of liver tumors. Int. J. Radiation Oncology Biol. Phys 57: 444–51. 10.1016/s0360-3016(03)00586-8 12957256

[7] Wulf J, Herfarth KK (2005) Normal tissue dose constraints in stereotactic body radiation therapy for liver tumors In: Kavanagh BD, Timmerman RD (eds). Stereotactic Body Radiation Therapy. Philadelphia: Lippincott Williams & Wilkins, 39–45.

[8] Sanuki-Fujimoto N, Takeda A, Ohashi T, Kunieda E, Iwabuchi S, Takatsuka T, et al (2010) CT evaluations of focal liver reactions following stereotactic body radiotherapy for small hepatocellular carcinoma with cirrhosis: relationship between imaging appearance and baseline liver function. BJR 83: 1063–71. 10.1259/bjr/74105551 21088090 PMC3473607

[9] Kimura T, Takahashi S, Kenjo M, Nishibuchi I, Takahashi I, Takeuchi Y, et al (2013) Dynamic Computed Tomography Appearance of Tumor Response after Stereotactic Body Radiation Therapy for hepatocellular Carcinoma-How should we evaluate treatment effects?-. Hepatol Res 43: 717–27. 10.1111/hepr.12007 23356835

[10] Kimura T, Hirokawa Y, Murakami Y, Tsujimura M, Nakashima T, Ohno Y, et al (2004) Reproducibility of organ position using voluntary breath-hold with spirometer for extracranial stereotactic radiotherapy. Int. J. Radiation Oncology Biol. Phys 60: 1307–13. 10.1016/j.ijrobp.2004.07.718 15519804

[11] Willemart S, Nicaise N, Struyven J, van Gansbeke D (2000) Acute radiation-induced hepatic injury: evaluation by triphasic contrast enhanced helical CT. BJR 73: 544–6. 10884753 10.1259/bjr.73.869.10884753

[12] Unger EC, Lee JKT, Weyman PJ (1987) CT and MR imaging of radiation hepatitis. J Comput Assist Tomogr 11: 264–8. 3819125 10.1097/00004728-198703000-00013

[13] Okumura T, Itai Y, Tsuji H, Matsueda K, Matsuzaki Y, Tsujii H (1994) Focused radiation hepatitis after bragg-peak proton therapy for hepatocellular carcinoma: CT findings. J Comput Assist Tomogr 18: 821–3. 8089336 10.1097/00004728-199409000-00025

[14] Guha C, Kavanagh BD (2011) Hepatic radiation toxicity: Avoidance and amelioration. Seminars in Radiation Oncology 21: 236–63. 10.1016/j.semradonc.2011.05.003 PMC3434677 21939854

[15] Olsen CC, Welsh J, Kavanagh BD, Franklin W, McCarter M, Cardenes HR, et al (2009) Microscopic and macroscopic tumor and parenchymal effects of liver stereotactic body radiotherapy. Int. J. Radiation Oncology Biol. Phys 73: 1414–24. 10.1016/j.ijrobp.2008.07.032 18990508

[16] Leen E, Goldberg JA, Anderson JR, Robertson J, Moule B, Cooke TG, et al (1993) Hepatic perfusion changes in patients with liver metastases: comparison with those patients with cirrhosis. Gut 34:554–7. 8491406 10.1136/gut.34.4.554 PMC1374320

[17] Reichen J, Egger B, Ohara N, Zeltner TB, Zysset T, Zimmermann A (1988) Determinants of hepatic function in liver cirrhosis in the rat. Multivariate analysis. J Clin Invest 82:2069–76. 3198765 10.1172/JCI113828 PMC442790

[18] Hashimoto K, Murakami T, Dono K, Hori M, Kim T, Kudo M, et al (2006) Assessment of the severity of liver disease and fibrotic change: the usefulness of hepatic CT perfusion imaging. Oncol Rep 16:677–83. 16969479

[19] Motosugi U, Ichikawa T, Sou H, Sano K, Tominaga L, Kitamura T, et al (2009) Liver parenchymal enhancement of hepatocyte-phase images in Gd-EOB-DTPA-enhanced MR imaging: Which biological markers of the liver function affect the enhancement? J Magn Reson Imaging 30:1042–6. 10.1002/jmri.21956 19856436

[20] Sanuki N, Takeda A, Oku Y, Eriguchi T, Nishimura S, Aoki Y, et al (2014) Threshold Doses for Focal Liver Reaction After Stereotactic Ablative Body Radiation Therapy for Small Hepatocellular Carcinoma Depend on Liver Function: Evaluation on Magnetic Resonance Imaging With Gd-EOB-DTPA. Int. J. Radiation Oncology Biol. Phys 88: 306–11. 10.1016/j.ijrobp.2013.10.045 24411601

