# Supplementary material for: The Time Course of Dynamic Computed Tomographic Appearance of Radiation Injury to the Cirrhotic Liver Following Stereotactic Body Radiation Therapy for Hepatocellular Carcinoma
Source: PLoS One. 2015 Jun 11;10(6):e0125231. doi: 10.1371/journal.pone.0125231 (PMC4466204; doi:10.1371/journal.pone.0125231)
Supplement: S1 Table — This table shows the clinical features, such as the Child–Pugh class, gender, age, total dose, PTV, tumor location (centrally located), history of resection, duration of initial treatment and adverse effects (Grade 3), according to type 1–3. Type 1, hyperdensity in all enhanced phasesType 2, hypodensity arterial, and portal phases, isodensity in venous phase and isodensity in the venous phaseType 3, isodensity in all enhanced phases. (PDF) [file pone.0125231.s001.pdf]

Table 1. Patient Background (77 patients with 92 HCCs)

**Age**	49–90 years (median, 71 years)	**Tumor size**	3–54 mm (median,19 mm)
**Gender**		**Tumor location**	
Male	49 patients	S1	2 lesions
Female	28 patients	S2	1 lesion
**Performance status (PS)**		S3	8 lesions
0	74 patients	S4	16 lesions
1	3 patients	S5	11 lesions
**Type of viral infection***		S6	9 lesions
HBV	6 patients	S7	19 lesions
HCV	62 patients	S8	26 lesions
NBNC	9 patients	**Previous treatment**	
**Child-Pugh class**		Surgery	29 patients
A	64 patients	RFA**	25 patients
B	13 patients	PEI[#]	12 patients
**Child-Pugh score**		TACE[$]	71 patients
5	48 patients		
6	16 patients		
7	8 patients		
≧8	5 patients		

Abbreviations: * HBV, hepatitis B virus; HCV, hepatitis C virus; NBNC, non-hepatitis B/ non-hepatitis C; HCC, hepatocellular carcinoma

** RFA, radiofrequency ablation

[#] PEI, percutaneous ethanol injection

[$] TACE, transcatheter arterial chemoembolization
