# Supplementary material for: The Time Course of Dynamic Computed Tomographic Appearance of Radiation Injury to the Cirrhotic Liver Following Stereotactic Body Radiation Therapy for Hepatocellular Carcinoma
Source: PLoS One. 2015 Jun 11;10(6):e0125231. doi: 10.1371/journal.pone.0125231 (PMC4466204; doi:10.1371/journal.pone.0125231)
Supplement: S2 Table — The title of Table 1 is “Patient Background (77 patients with 92 HCCs)”. This table shows the patients backgrounds, including age, gender, PS, type of viral infection, Child-Pugh class, Child-Pugh score, tumor size, tumor location and previous treatment. The title of Table 3 is “Univariate and Multivariate Analysis between the dynamic CT appearance of radiation injury to the liver and clinical features on Type 3 or Non-Type 3”. This table shows the results of univariate and multivariable analysis of the dynamic CT appearance of hepatic radiation injury for various time periods and the clinical features between type 3 or nontype 3 at 3–6 months, including the Child–Pugh class, gender, age, total dose, PTV, tumor location, history of resection, duration of initial treatment and adverse effects. The Child–Pugh class and adverse effects were significant factors in type 3 or nontype 3 (p < 0.0001, p = 0.003, respectively). In multivariable analysis, the Child–Pugh class was a only significant factor in type 3 or nontype 3 (p = 0.0005). (PDF) [file pone.0125231.s002.pdf]

Table 2. Background of dynamic CT appearance of radiation injury according to previous therapy

Previous therapies		3 months				3-6 months				6-12 months			
		Type 1	Type 2	Type 3	p-value	Type 1	Type 2	Type 3	p-value	Type 1	Type 2	Type 3	p-value
**TACE**	+	54	16	15	0.3176	68	11	6	0.0961	53	8	10	0.066
	-	4	1	2		7	0	0		7	0	0	
**Surgery**	+	24	6	8	0.4446	31	5	2	0.4765	25	3	6	0.2284
	-	34	11	9		44	6	4		35	5	4	
**Ablative therapies**	+	23	9	3	0.188	29	4	2	0.3938	25	4	2	0.2131
	-	35	8	14		46	7	4		35	4	8	

* TACE; transcatheter arterial chemoembolization
