# Supplementary material for: The Time Course of Dynamic Computed Tomographic Appearance of Radiation Injury to the Cirrhotic Liver Following Stereotactic Body Radiation Therapy for Hepatocellular Carcinoma
Source: PLoS One. 2015 Jun 11;10(6):e0125231. doi: 10.1371/journal.pone.0125231 (PMC4466204; doi:10.1371/journal.pone.0125231)
Supplement: S3 Table — The title of Table 2 is “Background of dynamic CT appearance of radiation injury according to previous therapy”. This table shows there were no significant difference in each enhancement pattern, such as type 1 to 3 in this study. (PDF) [file pone.0125231.s003.pdf]

Table 3. Univariate and Multivariate Analysis between the dynamic CT appearance of radiation injury to the liver and clinical features on Type 3 or Non-Type 3

		3- 6 months		P-value	P-value
		Type 3	Non-Type 3	Uni#	Multi##
**Child-Pugh class**	A	0	76	<0.0001	0.0005
	B	6	10		
**Gender**	male	5	53	0.2869	—
	female	1	33		
**Age**	>75	1	33	0.2869	—
	≦75	5	53		
**Total dose**	>48Gy	2	19	0.5259	—
	≦48Gy	4	67		
**PTV**	>25cc	1	35	0.2436	—
	≦25cc	5	51		
**Liver V20***	>10%	3	42	0.9561	—
**(Liver-PTV)**	≦10%	3	44		
**Tumor location**	periferal	4	70	0.3792	—
	central	2	16		
**History of**	+	2	36	0.6817	—
**resection**	—	4	50		
**Duration from**	>12 months	3	57	0.4182	—
**first treatment**	≦12 months	3	29		
**Adverse effects****	Grade 1 or 2	3	78	0.003	0.5666
	Grade 3	3	8		

Abbreviation: *V20: the percentage of the liver excluding PTV volume exceeding 20 Gy

** Adeverse effects were evaluated CTCAE ver.4.0.

uni: univariate analysis by the Mantel-Haenzel $\chi 2$ or t tests

Multi: univariate analysis by the Mantel-Haenzel $\chi 2$ or t tests
