# Supplementary figures and images for: The Time Course of Dynamic Computed Tomographic Appearance of Radiation Injury to the Cirrhotic Liver Following Stereotactic Body Radiation Therapy for Hepatocellular Carcinoma
Source: PLoS One. 2015 Jun 11;10(6):e0125231. doi: 10.1371/journal.pone.0125231 (PMC4466204; doi:10.1371/journal.pone.0125231)

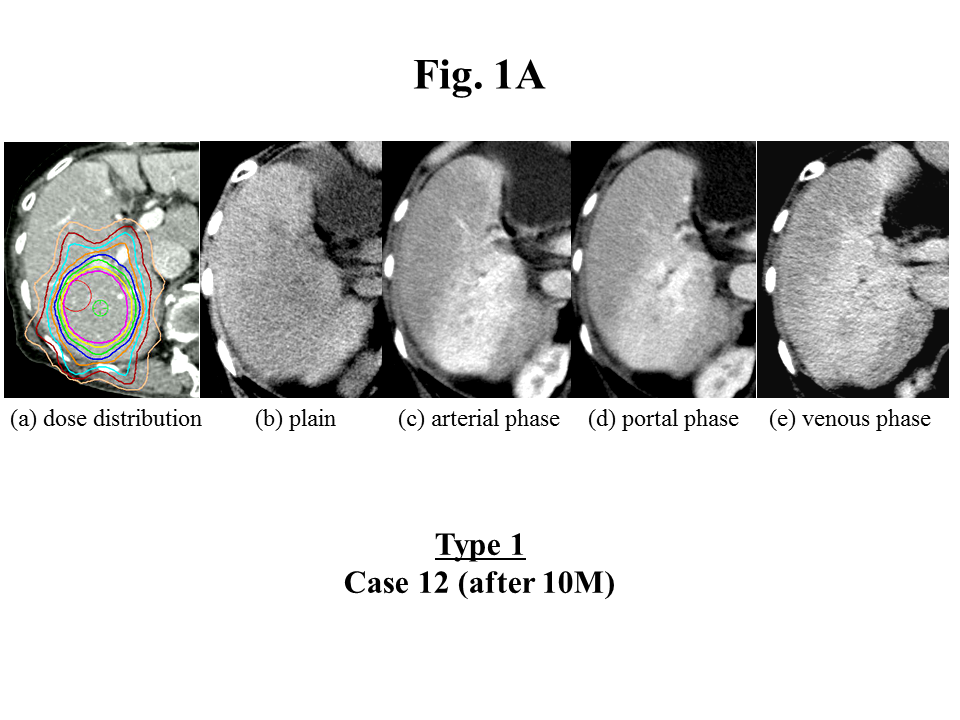

Supplement: S1 Fig — The title of Fig 1A is “The dynamic CT appearance of focal liver injury following SBRT for HCC was classified into 3 types. A) Type 1 (case 12, 10 months following SBRT)”. a) Dose distribution b) Plain c) Arterial phase d) Portal phase e) Venous phase. (TIF) [file pone.0125231.s004.tif]

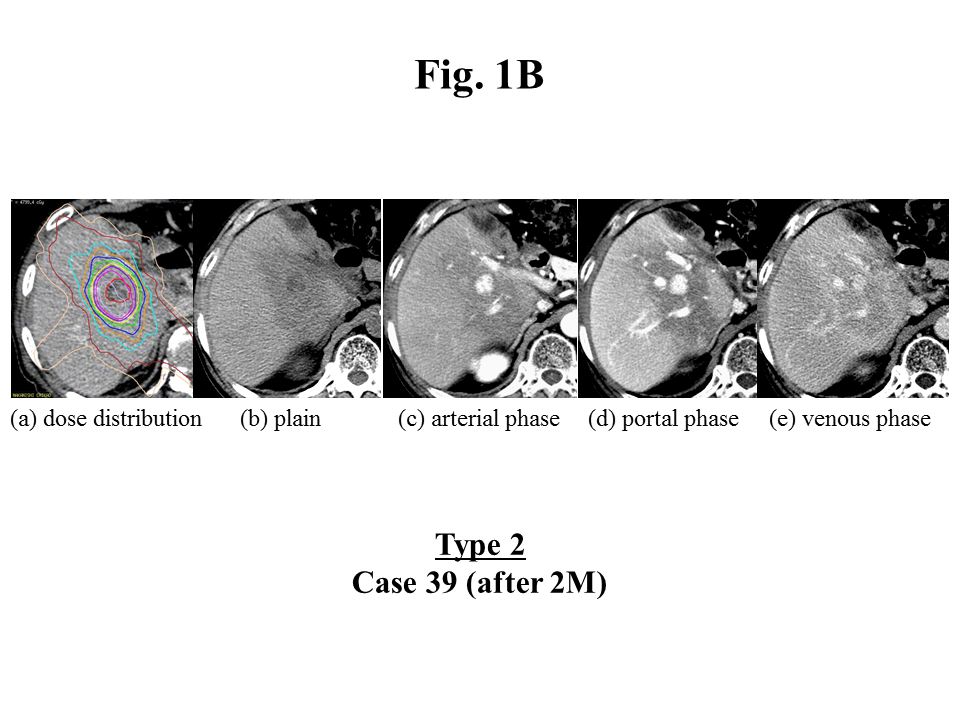

Supplement: S2 Fig — The title of Fig 1B is “The dynamic CT appearance of focal liver injury following SBRT for HCC was classified into 3 types. B) Type 2 (case 39, 2 months following SBRT)”. a) Dose distribution b) Plain c) Arterial phase d) Portal phase e) Venous phase. (TIF) [file pone.0125231.s005.tif]

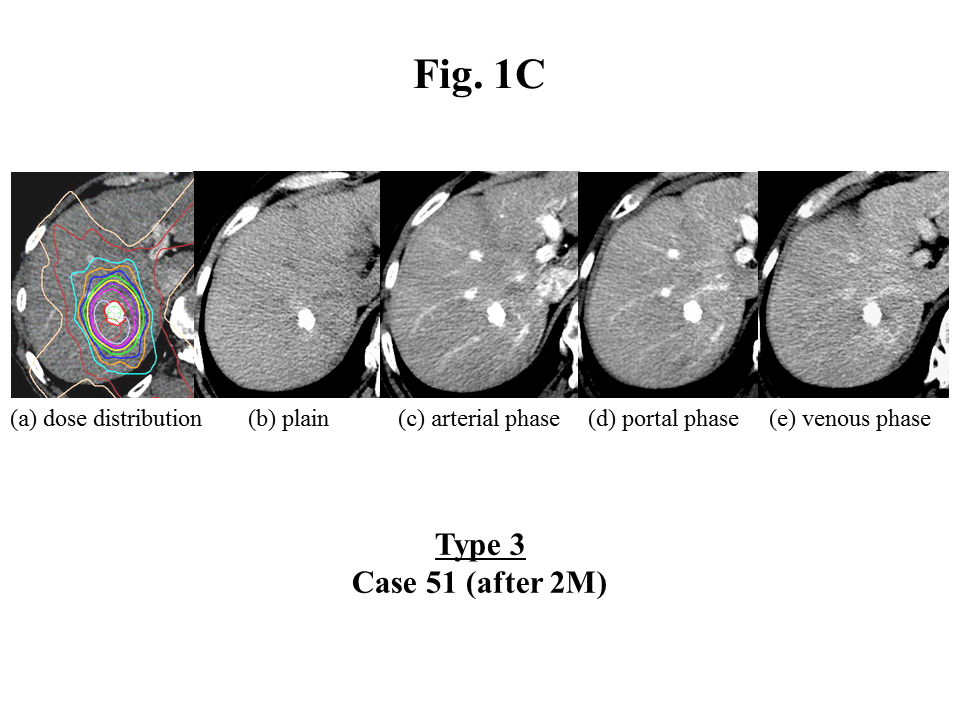

Supplement: S3 Fig — The title of Fig 1C is “The dynamic CT appearance of focal liver injury following SBRT for HCC was classified into 3 types. C) Type 3 (case 51, 2 months following SBRT)”. a) Dose distribution b) Plain c) Arterial phase d) Portal phase e) Venous phase. (TIF) [file pone.0125231.s006.tif]

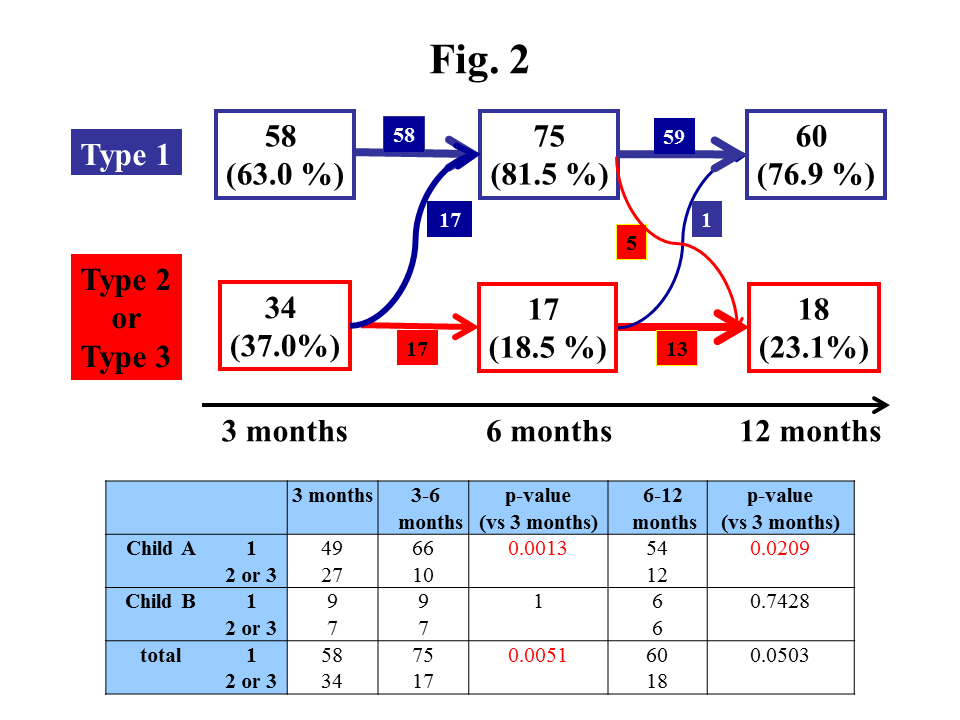

Supplement: S4 Fig — The title of Fig 2 is “The time course of dynamic CT appearance of radiation injury to liver according to Child-Pugh class”. The details of this figure was shown in Figure legends “Fig 2”. (TIF) [file pone.0125231.s007.tif]

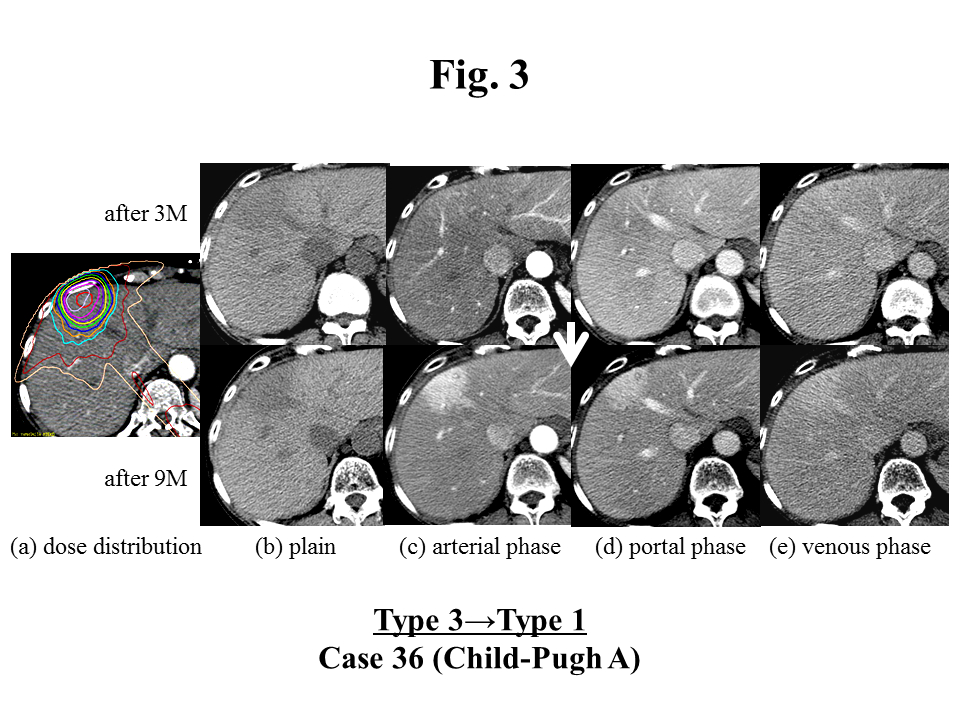

Supplement: S5 Fig — The title of Fig 3 is “A typical case belonging to Child–Pugh class A that changed from type 3 after 3 months to type 1 after 9 months (case 36).” Dose distribution b) Plain c) Arterial phase d) Portal phase e) Venous phase. (TIF) [file pone.0125231.s008.tif]
